# A Preliminary Investigation into the Use of Cannabis Suppositories and Online Mindful Compassion for Improving Sexual Function Among Women Following Gynaecological Cancer Treatment

**DOI:** 10.3390/medicina60122020

**Published:** 2024-12-07

**Authors:** Samantha Banbury, Hannah Tharmalingam, Joanne Lusher, Simon Erridge, Chris Chandler

**Affiliations:** 1 School of Psychology, London Metropolitan University, London N7 8DB, UK; chris.chandler@londonmet.ac.uk; 2 East and North Hertfordshire NHS Trust, Stevenage SG1 4AB, UK; hannah.tharmalingam@nhs.net; 3 Provost’s Group, Regent’s University London, London NW1 4NS, UK; 4 Medical Cannabis Research Group, Imperial College London, London SW7 2AZ, UK; simon.erridge12@imperial.ac.uk

**Keywords:** cancer, mindful compassion, mental health service delivery, quality of life

## Abstract

*Background and Objectives:* The impact of gynaecological cancer and its treatments on sexual intimacy can be profound on female sexuality. However, very few registered clinical trials have addressed sexual intimacy among this cohort. *Materials and Methods:* This preliminary randomised control trial (RCT) and content analysis assessed the effectiveness of a brief online mindful compassion group intervention adjunct with cannabis suppositories. Eighty-three participants aged between 18 and 50+ years who were at least six months post-cancer treatment were randomly allocated to one of four groups, depending on whether they were already using cannabis suppositories. These included a cannabis-only group (CO), a mindful-compassion group (MC), a combined mindful-compassion and cannabis suppositories group (COCM) and a care-as-usual group (CAU). Measurements of sexual function, sexual self-efficacy, mindful compassion, well-being and quality of life were taken at weeks 0, 4 and 12. *Results:* Sexual function, including arousal, lubrication and orgasms, improved for both the MC *p* = 0.002 and COCM *p* ≤ 0.001 groups; in addition, sexual pain was reduced in the COCM *p* = 0.008 and CO *p* ≤ 0.001 groups compared to the CAU and MC groups, where *p* ≥ 0.05. Feedback suggested that cannabis mediated the effects of mindful compassion and supported well-being, sexual self-efficacy, and quality of life. Participants also voiced a preference for cannabis suppositories when using dilators as part of their treatment and the use of sex toys instead of dilators, suggesting that dilators had negatively impacted their sexuality. *Conclusions:* These preliminary and exploratory outcomes look promising and provide a foundation for future research to develop varied healthcare options to improve mental health service delivery and quality of life for this cohort.

## 1. Introduction

There are five main types of gynaecological cancers (GC): ovarian, uterine, vulvar, cervical, and vaginal. In the UK, there are approximately 20,000 annual diagnoses of one of these cancers [1]. According to research [2], 1.39 million women live with GC. The diagnosis of GC negatively impacts psychological well-being, sexual well-being and quality of life [3]. Most of those with GC present with sexual dysfunction [4,5]. Briefly, sexual dysfunction is a group of sexual disorders in which there are disruptions in the sexual response cycle, including the loss of one or more of the phases or aspects of sexual response, i.e., sexual desire, sexual arousal, vaginal lubrication, orgasm, and sexual pain [6]. This tends to include up to 75% of sexual attempts and can be lifelong or acquired in presentation [6].

Sexual functioning can be impaired by the treatments; these often involve pelvic irradiation, the long-term side effects of which can impact lubrication and orgasm and cause pain and arousal difficulties [7,8]. The impact of gynaecological brachytherapy, which is the standard of care for cervical cancer patients undergoing radical treatment, has frequently been reported to cause long-term pain, anxiety and distress [9]. Numbness/loss of sensation may be a further byproduct of treatments for GC, along with early menopause, further resulting in sexual dysfunction [8,10].

There is a lack of effective therapies for these individuals affected by sexual dysfunction, and various psychosexual interventions have been suggested [8]. The use of testosterone for women with compromised sexual desire remains inconclusive. In an RCT looking at the role that transdermal testosterone may play in sexual desire among post-breast cancer women, the results were non-significant [11]. The use of oestrogen at low doses for women with a history of gynaecological cancer and who are going through menopause has also been recommended [12]. Therapeutically, the use of cognitive behavioural therapy (CBT) looks favourable in the context of supporting sexual dysfunction. A meta-analysis yielded a moderate effect size (d = 0.58) when using CBT to support sexual dysfunction [13]. Of a similar effect size, a meta-analysis looking at mindfulness training has shown it to support sexual dysfunction (d = 0.55) [14]. Other authors [15] compared CBT with mindfulness training for sexual pain and found that mindfulness was more effective than CBT. A systematic review of 10 studies looking at sexual intimacy and cancer found that mindfulness was supportive in helping women with sexual dysfunction who were post-breast or gynaecological cancer across all sexual domains [16]. In one such study [7], 31 participants with cervical cancer (mean age 54.0; age range 31–64) with sexual desire and/or sexual arousal concerns were randomised into an experimental group consisting of three 90-min mindfulness-based cognitive behaviour therapy sessions or a wait-list control group (participants would receive the intervention after 2 months). Outcomes led to significant improvements in sexual domains, including sexual desire and arousal, with a reduction in sexual distress.

The use of mindful compassion has now been introduced to supporting women post-breast cancer treatment. Briefly, mindful compassion focuses on embracing self-compassion and accepting negative feelings and thoughts. This method uses mindfulness in a more targeted way to support emotional discomfort with compassion and acceptance [17]. A few studies have looked at the role that mindful compassion might have in supporting sexual dysfunction. For example, in a sample of 36 women with GPPPD (genito-pelvic pain/penetration disorder), the technique was found to be supportive in reducing sexual pain after six mindful compassion workshops [18]. Our unpublished preliminary data provided a brief online mindful compassion intervention for 52 women post-breast cancer treatment in a waitlist-controlled randomised controlled trial (RCT). This comprised a brief 4-week online intervention. The outcomes were promising, and improvements were reported across all sexual domains, including sexual arousal, sexual desire, orgasm, satisfaction and lubrication. However, orgasms fluctuated across the delivery of this intervention. A feedback evaluation by participants suggested that they would have preferred their partners to be included in the study. Whilst their sexual desire and arousal had increased, their partners’ levels had remained the same, resulting in a sexual desire discrepancy. Further, the topic of sexual intimacy was not adequately addressed by healthcare practitioners, which had impacted quality of life (QOL). Indeed, out of 42 participant responses yielded from the content analysis, 31% stated that “we are human and have needs”, and 11.9% felt like they had been shut down in healthcare when the topic of sex was raised and were offered support. Despite research highlighting the relationship between GC and sexual difficulties, there is a paucity of research looking at suitable interventions to support the psychosexual elements associated with sexual dysfunction in oncology healthcare settings [19].

Cannabis has also been used historically to address sexual pain. For example, a systematic review of 16 studies revealed that the use of CBD or THC at between 70 and 2000 mg significantly reduced levels of gynaecological pain in up to 61–95.5% of participants after 3 months of use [20]. In the UK, medical cannabis (MC) was rescheduled in 2018 and can now be prescribed for individuals with chronic physical and mental health conditions that are refractory to licensed medications [21]. Research, although inconclusive, has suggested that cannabis possesses analgesic properties and can help reduce pain [22]. Limited research has compared the pain-minimising properties of CBD and THC suppositories. However, researchers comparing the pain-minimising effects of THC suppositories compared to the oral use of THC have suggested that their efficacy is up to 50% higher [23].

Mindful compassion and cannabis suppositories have been suggested in supporting sexual pain among men who have sex with men (MSM) with anodyspareunia [24]. A sample of 52 MSM was randomised to one of four groups, based on whether they had been using cannabis suppositories. This included THC, CBD and combined CBD/THC. Groups included a mindful-compassion-only group, a cannabis suppository-only group, a combined mindful compassion and cannabis suppository group and a care-as-usual group. Measurements of anodyspareunia, mindful compassion, well-being, sexual functioning and sexual self-efficacy were taken at weeks 0, 4 and 12. The outcomes favoured the combined cannabis suppository and mindful compassion group compared to the other groups in all domains. However, there was no significant difference between outcomes in terms of pain regarding the use of CBD, THC and combined THC/CBD suppositories. The authors attributed this to the small sample size, which means that a larger sample might have yielded different outcomes.

Based on the literature review, it was decided to explore the use of mindful compassion with cannabis suppositories for women after gynaecological cancer treatment. To the authors’ knowledge, this is the first study to address this adjunct intervention among this cohort. However, randomisation was limited owing to the legalities of medical cannabis in the UK; hence, the cohort was randomised into groups based on whether they were using cannabis suppositories. We also wanted to establish whether the outcomes would indicate any differences between THC and CBD in terms of levels of pain. As well as pain, other sexual domains including lubrication, arousal, desire, orgasm and sexual satisfaction were measured, along with well-being, sexual self-efficacy and QOL. For this preliminary study, the focus was on vaginal intercourse and masturbation only (not anal sex).

The following four groups were included in this study.

(1) Cannabis-only group (CO).
(2) Mindful compassion group (MC).
(3) Cannabis suppositories and mindful compassion group adjuncts (COMC).
(4) Care as usual (CAU/control group/not using cannabis suppositories or engaging in mindful compassion).

Randomisation to the mindful compassion or CAU groups depended on whether participants were using cannabis suppositories. The following hypotheses were assessed:

- There would be a significant effect of time on sexual self-efficacy, mindful compassion, sexual functioning, well-being and QOL for the CO, MC and COMC groups.
- There would be no significant effect of time on sexual self-efficacy, mindful compassion, sexual functioning, well-being and QOL in the CAU group.
- Levels of sexual functioning and sexual pain would vary between CBD and THC suppositories in the MC and COMC groups.

## 2. Materials and Methods

### 2.1. Design

#### 2.1.1. Study 1

**Figure 1:**
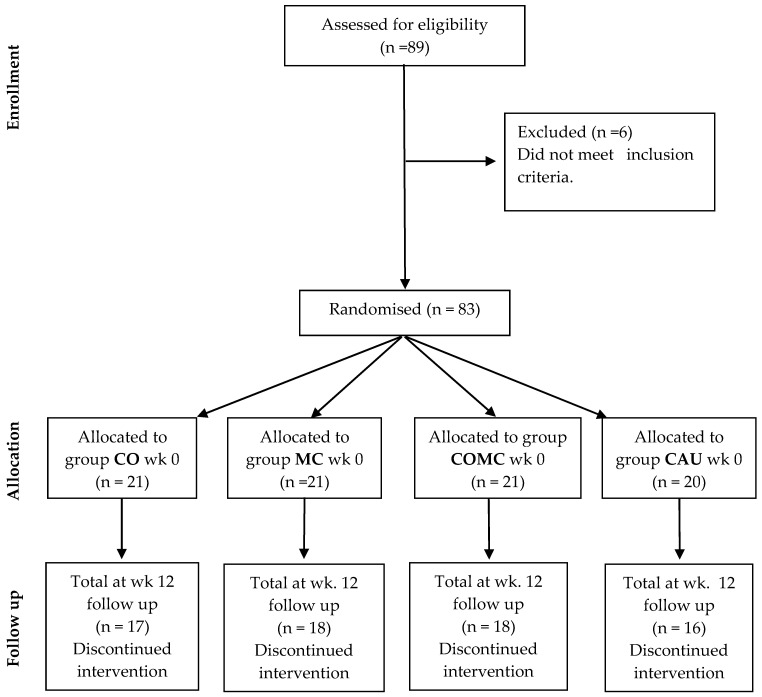
The flow of participants through each stage of a combined cannabis suppository and mindful compassion randomised controlled trial. Key: Cannabis-only group (CO), mindful compassion group (MC), cannabis suppositories and mindful compassion group adjuncts (COMC), and care as usual (CAU).

A parallel randomisation approach was used with four groups: the CO, MC, COMC and CAU comparative groups. The intervention (see Figure 1) was delivered online for four weeks, with a twelve-week follow-up. Participants were sourced via TikTok and LinkedIn.

#### 2.1.2. Study 2

Feedback, gathered using the Mentimeter app (Mentimeter, Stockholm, Sweden), was sought throughout the intervention’s delivery at weeks 0, 4 and 12. A series of open-ended questions was aimed at establishing the participants’ thoughts and feelings about the intervention. The questions were similar for all groups; however, the questions had to apply to each group and were based on the questions used in a previous study [24], which examined MSM with anodyspareunia.

### 2.2. Participants

As can be seen in Table 1, Eighty-three participants contributed to the study (n = 21 CO, n = 21 MC, n = 21 COMC, n = 20 CAU). Forty-two participants were already using cannabis suppositories. Attrition rates were reasonable and predominated in the CAU and CO groups, with 69 (83.1%) remaining in the study at week 12 of follow-up (n = 17 CO, n = 18 MC, n = 18 COCM, n = 16 CAU). Eleven participants (13.3%) were aged between 18 and 30 years; n = 51 (61.4%) were aged between 31 and 50 years and 21 participants (25.3%) were aged 51 years and above. Menopause, including early menopause, accounted for n = 43 (51.8%) participants. Ethnicity consisted of n = 52 (62.7%) white, n = 30 (36.1%) African Caribbean and n = 1 (1.2%) Pakistani participants. Of the sample, n = 21 (25.4%) were not partnered, n = 7 (8.4%) were partnered for up to 1 year, n = 15 (18.1%) had been partnered for between 1 and 2 years, n = 11 (13.3%) were partnered up to 4 years and n = 29 (34.9%) were partnered for 5 years and above. Of these, n = 77 (92.8%) identified as heterosexual and n = 6 (7.2%) were bisexual. Outside of post-cancer treatments, n = 44 (53.0%) were not taking any prescription medication, n = 26 (31.3%) were prescribed heart medication and n = 13 (15.7%) were using insulin. Outside the use of cannabis suppositories, n = 53 (63.9%) were not using any substances, n = 7 (8.4%) used cocaine recreationally and n = 4 (4.8%) used speed/amphetamine. N = 23 (27.7%) did not drink alcohol, n = 49 (59.0%) consumed up to 14 units of alcohol weekly, and n = 11 (13.3%) consumed more than 14 units of alcohol weekly. Of the sample, n = 42 (50.6%) did not exercise, n = 15 (18.1%) exercised approximately 3 times a week, n = 18 (21.7%) exercised weekly and n = 8 (9.6%) did not state their exercise level. Post-cancer duration included 6 months (n = 25 (30.1%)), 12 months (n = 23 (27.7)), 18 months (n = 23 (27.7%)) and 24 months (n = 12 (14.5%)). The stages of cancer varied from stage 1 (n = 41 (49.4%)), stage 2 (n = 39 (47.0)) to stage 3 (n = 3 (3.6%)). Types of gynaecological cancer included n = 43 (51.8%) uterine, n = 30 (36.1%) cervical, n = 7 (8.4%) vaginal and n = 3 (3.6%) vulval cancer. Cancer treatment involved surgery (n = 8 (9.6%)), radiotherapy (n = 9 (10.8%)), chemotherapy (n = 8 (9.6%)), combined radiotherapy, chemotherapy and hormones (n = 38 (45.8%)), hormones (n = 14 (16.9%)) and targeted therapy (n = 6 (7.2%)). The main reported reason for using cannabis suppositories involved sexual pain during intercourse (n = 9 (10.8%)), assisting with the use of dilators (n = 8 (9.6%)) and combined sexual pain and dilators (n = 25 (30.1%)), with n = 41 (49.4%) reporting it as not applicable. N = 15 (21.7%) participants used oestrogen gel during sexual intimacy. Among those using THC suppositories, n = 37 (44.6%) had acquired a formal prescription but reformulated it themselves into a suppository. Suppositories were mainly used every 2 weeks (n = 22 (26.5%)), weekly (n = 13 (15.7%)) and more than weekly (n = 12 (14.5%)). The duration of vaginal insertion of the cannabis suppositories before sexual intercourse was n = 20 (24.1%)—30 min beforehand, n = 18 (21.7%)—between 30 and 60 min, n = 4 (4.8%)—over 60 min, with n = 41 (49.4%) reporting that it was not applicable. Dosages varied; for n = 11 (13.3%) it was 100 mg, for n = 10 (12.0%), 500 mg, for n = 16 (19.3%), 1000 mg and n = 5 (6.0%) were unsure, with n = 41 (49.4%) reporting it as not applicable. No condoms were used among n = 62 (74.7%) of the cohort, n = 12 (14.5%) used regular condoms (waiting for the oil to dissipate), and n = 9 (10.8%). Use of THC suppositories was reported in n = 12 (14.5%), CBD suppositories, in n = 11 (13.3%) and combined THC/CBD suppositories, in n = 19 (22.9%), with n = 41 (49.4%) reporting it as not applicable.

The following exclusion criteria formed part of this study.

#### 2.2.1. Inclusion Criteria

- Participants should be at least six months or more post-cancer treatment (excluding hormone treatment).
- If applicable, each participant had been using cannabis (THC, CBD, THC/CBD adjunct) suppositories for at least one month.
- Participants should be registered with a general practitioner (GP)
- Sexual functioning involving vaginal sex was satisfactory before cancer diagnosis (acquired).
- Participants were attempting sexual intimacy.
- Participants were aged 18 years or older.
- Participants were fluent in reading and writing English (as this is a clinical trial, we wanted to ensure that participants fully understood what was expected of them).
- A patient health questionnaire-PHQ-9 score of between 0 and 9 [25].

#### 2.2.2. Exclusion Criteria

- Applicants were currently receiving cancer treatment, such as radiation therapy or chemotherapy.
- Applicants were not registered with a GP.
- Applicants were sexually abstinent.
- Applicants were aged under 18 years old.
- Sexual functioning involved anal sex
- Applicants showed difficulties in reading and writing English.
- Applicants had lifelong sexual function difficulties.
- Applicants had a PHQ-9 score range between 10 and 27.

### 2.3. Mindful Compassion Intervention

Mindful compassion exercises are intended not only to support increased sexual well-being and sexual self-efficacy [26] but also to manage sexual pain. The main exercises included mindfulness, breathing, relaxation techniques and body scans. These exercises incorporate the three-model system of emotions and how to attend to the cognitive and physiological patterns associated with sexual functioning. This intervention was based on the behavioural taxonomy of selected behaviour change techniques (BCTTv1) [27]. The intervention was adapted for this study but was previously tested among different mindfulness cohorts [16,24,28].

### 2.4. Self-Report Measures

#### 2.4.1. Demographic Information

Demographic information included gynaecological cancer, stage of cancer, cancer treatments used, engagement in vaginal or anal sex, sexual difficulties, partnered status, ethnicity, sexuality, age, type of cannabis use (THC, CBD, none), frequency of cannabis suppository use, prescription medication, exercise use, alcohol consumption and illicit drug use.

#### 2.4.2. Patient Health Questionnaire (PHQ-9) [25]

The PhQ-9 [25] was used to screen for depression. The internal reliability was within the range of 0.86–0.89, which indicates good reliability. The 9-item measure requests participants to rate the regularity of present difficulties during the past 2 weeks (e.g., “Trouble falling or staying asleep or sleeping too much”). Scores indicate the presence and the severity of the depression, with a maximum score of 27 and a minimum score of 0.

#### 2.4.3. The Female Sexual Function Index (FSFI) [29]

The FSFI [29] is a 19-item measure of sexual pain, sexual desire, orgasm, lubrication and sexual satisfaction, with five response categories. Example questions include: “Over the past 4 weeks, how often did you experience discomfort or pain during vaginal penetration?” and “Over the past 4 weeks, how would you rate your level (degree) of discomfort or pain during or following vaginal penetration?”; Cronbach’s α = 0.820 and higher. In the present study, Cronbach’s α = 0.750.

#### 2.4.4. Adapted Sexual Self-Efficacy Scale for Female Sexual Functioning (SSES-F) [30]

This is a 37-item measure that embraces aspects of female sexuality such as arousal, desire, orgasm, pain and satisfaction. The SSES-F [30] has 10 response categories. Example questions include anticipating (thinking about) having intercourse without fear or anxiety and engaging in intercourse without pain or discomfort; Cronbach’s α ≥ 0.930. In the present study, Cronbach’s α = 0.852.

#### 2.4.5. The Short Warwick-Edinburgh Mental Well-Being Scale (SWEMWBS) [31]

This is a seven-item questionnaire with five response categories, looking at functioning and feeling aspects of well-being. An example question includes: “I’ve been feeling relaxed”. In the present study, Cronbach’s α = 0.834.

#### 2.4.6. Brunnsviken Brief Quality of Life Scale (BBQ) [32]

This is an eight-item questionnaire with five response categories, looking at satisfaction with self, friends, family and creativity. Example responses include “how I view my life as necessary for my quality of life”, and “I am satisfied with my friends and friendship”; Cronbach’s α = 0.760. In the present study, Cronbach’s α = 0.789.

#### 2.4.7. State Self-Compassion (with Mindfulness) Short Form [33]

This is a 12-item measure with 5 response categories, where 1 = almost never, to 5 = almost always, with higher scores indicating higher levels of self-compassion. The questionnaire measures self-kindness vs. self-judgement, common humanity vs. isolation, and mindfulness vs. over-identification with painful thoughts and emotions. Reliability scores for Cronbach’s alpha ranged between 0.680 and 0.780.

#### 2.4.8. Feedback Questions

Mentimeter (Mentimeter, Stockholm, Sweden) feedback questions were asked throughout this study, as part of the content analysis. The responses from week 12 have been provided. The questions used were sourced from a prior study examining sexual pain among men who have sex with men (MSM) and who experience anodyspareunia [23]. The responses deemed relevant to this cohort in future healthcare planning have been included to avoid repetition.

### 2.5. Procedure

The intervention was registered with clinicaltrials.gov, NCT06607835, and was ethically approved by a university ethics review panel. This study was conducted under the British Psychological Society (BPS) Code of Ethics and Conduct [34]. Details of the study were made available online via LinkedIn and TikTok. The PHQ-9 was used as a screening tool for this study’s inclusion and exclusion criteria. Eligible consenting participants were randomly allocated to a group, based on whether they were using cannabis suppositories. This criterion was chosen owing to the legalities associated with cannabis in the UK. All participants completed baseline assessments at week 0. This was repeated at weeks 4 and 12. The ordering used for the online Microsoft survey (Microsoft^®^, Redmond, WA, USA) was as follows: The Female Sexual Function Index; Adapted Sexual Self Efficacy Scale for Female Sexual Functioning; Short Warwick–Edinburgh Mental Wellbeing Scale; Brunnsviken Brief Quality of Life Scale and State Self-compassion Scale, followed by the debrief, including additional support services and a complaints contact form. Feedback on the intervention was sought throughout this study using Mentimeter (Mentimeter, Stockholm, Sweden) at weeks 0, 4 and 12. The online delivery of the mindful compassion intervention was conducted over 4 weeks (1 h per session) for the MC and COMC. Data were stored in OneDrive (Microsoft^®^, WA, USA), based at the University, and were managed according to the Data Protection Act (2018).

### 2.6. Statistical Analysis

A Cronbach’s alpha analysis was conducted on the assessments included in this study.

A repeated-measures ANOVA on sexual pain, sexual function, mindful compassion, quality of life and well-being (IV) compared the means of these variables at 0, 4, and 12 weeks (follow-up) for all groups. A post hoc test using Friedman’s two-way ANOVA K samples was conducted on statistically significant outcomes. An ANOVA determined the differences in these variables between all groups. Furthermore, a MANOVA compared the use of THC, CBD and a combination of THC/CBD with levels of vaginal sexual functioning and pain. A subsequent MANCOVA explored this finding further but controlled for the dosages of cannabis suppository use. A nonparametric content analysis was conducted on the collated feedback from participants at weeks 0, 4 and 12. Week 12 has been included in this study. The effect size was partial eta squared, combined with 95% confidence intervals (95% CI). For the content analysis, it was decided to include only week 12’s feedback to establish the intervention’s ongoing outcomes and report the most significant/highest outcomes, as these would inform the development of this intervention. Statistical analysis was conducted using SPSS version 28.0 [IBM, Armonk, NY, USA].

## 3. Results

### 3.1. The Impact of Time on Mindful Compassion, Sexual Functioning, Sexual Self-Efficacy, Well-Being and Quality of Life

Regarding the CO group, there was an effect of time on sexual functioning at weeks 0 to 12, where F(2, 15) = 12.933, *p* ≤ 0.001, *η_p_*^2^= 0.633, CI: 18.356–23.64. Among the sexual functioning variables, including sexual desire, sexual arousal, lubrication, orgasm, sexual satisfaction and sexual pain, sexual pain was significant between weeks 0 and 12, where F(2, 15) = 11.239, *p* ≤ 0.001, *η_p_*^2^ = 0.600, CI: 7.006–4.630. The remaining sexual functioning variables were non-significant, where *p* > 0.05. There was an effect of time on well-being at weeks 0 to 12, where F(1, 16) = 21.361, *p* ≤ 0.001, *η_p_*^2^ = 0.572, CI: 17.505–21.319. There was an effect of time on sexual-self efficacy, where F(2, 15) = 8.190, *p* = 0.004, *η_p_*^2^ = 0.522, CI: 13.759–15.758. There was no effect on time for mindful compassion or QOL, at *p* > 0.05. Post hoc tests for sexual functioning, sexual pain, well-being and sexual self-efficacy were significant, at *p* < 0.05. There was an overall interaction effect with sexual function and well-being, mindful compassion, sexual self-efficacy and QOL, where F(7, 10) = 175.582, *p* < 0.001, *η_p_*^2^ = 0.996, CI: 4.630–21.319. Post hoc tests across all variables were significant, at *p* < 0.001.

Concerning the MC group, there was an effect of time on sexual functioning at weeks 0 to 12, where F(2, 16) = 9.394, *p* = 0.002, *η_p_*^2^ = 0.540, CI: 20.543–25.471. Among the sexual functioning variables, including sexual desire, sexual arousal, lubrication, orgasm, sexual satisfaction and sexual pain, sexual arousal was significant between weeks 0 and 12, where F(2, 16) = 5.757, *p* = 0.013, *η_p_*^2^ = 0.419, CI: 3.261–4.998; lubrication was significant between weeks 0 and 12, where F(1, 17) = 5.591, *p* = 0.013, *η_p_*^2^ = 0.247, CI: 3.621–5.101 and orgasms were significant between weeks 0 and 12, where F(2, 16) = 4.382, *p* = 0.030, *η_p_*^2^ = 0.354, CI: 1.453–3.144. Sexual desire, sexual satisfaction and sexual pain were non-significant at *p* > 0.05. There was an effect of time on well-being at weeks 0 to 12, where F(2, 16) = 46.241, *p* ≤ 0.001, *η_p_*^2^ = 0.853, CI: 15.413–23.781; sexual self-efficacy, F(2, 16) = 61.517, *p* ≤ 0.001, *η_p_*^2^ = 0.885, CI: 14.234–26.897; mindful compassion, F(2, 16) = 34.910, *p* ≤ 0.001, *η_p_*^2^ = 0.814, CI: 21.256–34.859 and QOL, F(2, 16) = 3.954, *p* = 0.04, *η_p_*^2^ = 0.331, CI: 15.291–20.646. Post hoc tests on sexual functioning, sexual arousal, lubrication, orgasm, sexual self-efficacy, mindful compassion and QOL were significant at *p* < 0.05. There was an overall interaction effect with sexual function and well-being, mindful compassion, sexual self-efficacy and QOL, where F(4, 14) = 66.340, *p* < 0.001, *η_p_*^2^ = 0.950, CI: 20.219–25.471. Post hoc tests for sexual function and mindful compassion and QOL were significant at *p* < 0.001, but not significant for sexual function and well-being and sexual self-efficacy, at *p* > 0.05.

Regarding the COMC group, there was an effect of time on sexual functioning at weeks 0 to 12, where F(2, 16) = 20.792, *p* ≤ 0.001, *η_p_*^2^ = 0.722, CI: 20.543–28.787. Among the sexual functioning variables, including sexual desire, sexual arousal, lubrication, orgasm, sexual satisfaction and sexual pain, sexual arousal was significant between weeks 0 and 12, where F(2, 16) = 9.780, *p* = 0.002, *η_p_*^2^ = 0.550, CI: 3.261–5.224; lubrication was significant between weeks 0 and 12, where F(1, 17) = 13.600, *p* = 0.002, *η_p_*^2^ = 0.444, CI: 3.822–5.512; orgasms were significant between weeks 0 and 12, where F(2, 16) = 8.063, *p* = 0.004, *η_p_*^2^ = 0.502, CI: 1.453–3.771 and sexual pain was significant (reduced) between weeks 0 and 12, where F(2, 16) = 6.694, *p* = 0.008, *η_p_*^2^ = 0.456, CI: 7.084–5.360. Sexual desire and sexual satisfaction were non-significant at *p* > 0.05. There was an effect on time on well-being at weeks 0 to 12, where F(2, 16) = 70.998, *p* ≤ 0.001, *η_p_*^2^ = 0.899, CI: 12.364–25.270; sexual self-efficacy, F(2, 16) = 63.821, *p* < 0.001, *η_p_*^2^ = 0.889, CI: 14.230–28.717; mindful compassion, F(2, 16) = 37.984, *p* < 0.001, *η_p_*^2^ = 0.826, CI: 21.256–39.517 and QOL, F(2, 16) = 4.975, *p* = 0.021, *η_p_*^2^ = 0.383, CI: 15.219–20.646. Post hoc tests on sexual functioning, sexual arousal, lubrication, orgasm, sexual pain, sexual self-efficacy, mindful compassion and QOL were all significant at *p* < 0.05. There was an overall interaction effect with sexual function and well-being, mindful compassion, sexual self-efficacy and QOL, where F(4, 14) = 58.483, *p* < 0.001, *η_p_*^2^ = 0.944, CI: 22.285–28.787. Post hoc tests for sexual function and well-being were significant, at *p* > 0.005, and for mindful compassion and QOL, *p* > 0.001, but not for sexual self-efficacy, at *p* > 0.005.

Regarding the CAU group, there was an effect of time on well-being at weeks 0 to 12, where F(2, 14) = 11.974, *p* ≤0.001, *η_p_*^2^ = 0.527, CI: 23.612–19.174 and sexual self-efficacy, where F(2, 14) = 13.455, *p* = 0.004, *η_p_*^2^ = 0.383, CI: 15.219–20.646. Post hoc tests revealed significance in the diminishing levels of well-being and sexual self-efficacy, at *p* > 0.05. There was no effect on time on the sexual function variables, mindful compassion or QOL, at *p* > 0.05. However, the QOL value was borderline. There was an overall interaction effect, where F(4, 12) = 19.375, *p* < 0.001, *η_p_*^2^ = 0.866, CI: 15.826–18.360. Post hoc tests on sexual self-efficacy were non-significant. Interestingly, QOL decreased at *p* = 0.022, and well-being at *p* < 0.001.

### 3.2. Comparisons Across Groups

When comparing across the groups, CAU, CO, MC, and COMC, for well-being at week 12, F(3, 13) = 11.486, *p* < 0.001, *η_p_*^2^ = 0.726, CI: 17.190–25.452. Post hoc tests on CAU with CO were non-significant, at *p* > 0.05, but were significant with MC, at *p* = 0.012 and COMC, at *p* < 0.001. Additionally, significant outcomes were observed between CO and COMC, at *p* < 0.05. There were no further significant post hoc outcomes. When comparing mindful compassion groups, F(3, 13) = 202.624, *p* < 0.001, *η_p_*^2^ = 0.979, CI: 19.025–40.028. Post hoc tests on CAU with CO, MC and COMC were significant, at *p* < 0.001. There was no significant difference between MC and COMC, at *p* > 0.05. When comparing groups’ sexual self–efficacy at week 12, F(3, 13) = 41.657, *p* < 0.001, *η_p_*^2^ = 0.906, CI: 15.506–27.980. Post hoc tests on CAU with CO were significant, at *p* > 0.05, and significant with MC and COMC, at *p* < 0.001. Additionally, significant outcomes were observed for CO and MC and CO with COMC, at *p* < 0.001. When comparing sexual functioning among the groups, F(3, 13) = 53.265, *p* < 0.001, *η_p_*^2^ = 0.925, CI: 18.306–29.240. Post hoc tests were significant for CAU and all groups, at *p* < 0.001. Additional significant outcomes were found with CO and MC and with MC and COMC, at *p* ≥ 0.05, along with CO and COMC, at *p* < 0.001. Sexual arousal was non-significant across groups, where F(3, 13) = 2.381, *p* = 0.117, *η_p_*^2^ = 0.355, CI: 4.885–5.262. Lubrication was significant across groups, where F(3, 13) = 11.839, *p* < 0.001, *η_p_*^2^ = 0.732, CI: 3.493–5.635. Post hoc tests for CAU with CO and MC were significant, at *p* > 0.05, and significant for COMC, at *p* < 0.001. A significant outcome was observed with CO and COMC, at *p* ≤ 0.05. Orgasms were non-significant across groups, where F(3, 13) = 2.517, *p* ≥ 0.05, *η_p_*^2^ = 0.367, CI: 2.955–4.018. Sexual satisfaction was significant across groups, where F(3, 13) = 11.854, *p* < 0.001, *η_p_*^2^ = 0.732, CI: 3.294–5.674. Post hoc tests for CAU with MC were significant, at *p* > 0.05, and for COMC, at *p* < 0.001. An additional significant outcome was observed with MC and COMC, at *p* < 0.001. Sexual pain was significant across groups, where F(3, 13) = 18.984, *p* < 0.001, *η_p_*^2^ = 0.814, CI: 3.335–7.596. Post hoc tests for CAU with CO were significant, at *p* < 0.05, and MC and COMC at *p* < 0.001. Additional significant outcomes were CO and MC with COMC, at *p* < 0.001 and MC and COMC, at *p* < 0.05. QOL was non-significant across groups at week 12, where F(3, 13) = 0.925, *p* = 0.420, *η_p_*^2^ = 0.117, CI: 12.572–17.575.

### 3.3. Sexual Functioning with the Use of THC and CBD Suppositories

There was a statistically significant interaction effect between the doses of THC and CBD suppositories and how often these were used in the COMC group than the CO group for sexual satisfaction, where F(1, 16) = 18.375, *p* = 0.023; *η_p_*^2^ = 0.860. There was an interaction effect between the THC/CBD dose and duration of use before sexual functioning in the COMC than the CO group for overall sexual functioning, where F(1,16) = 11.344, *p* = 0.044; *η_p_*^2^ = 0.791; for sexual satisfaction, F(1, 16) = 18.000, *p* = 0.024, *η_p_*^2^ = 0.857 and for sexual pain, F(1, 16) = 11.605, *p* = 0.042, *η_p_*^2^ = 0.795. For sexual functioning in the COMC group, post hoc tests were significant for combined THC/CBD use at 1000 mg, at *p* < 0.05, and approximately 30 min before intimacy for sexual pain, at *p* < 0.05. When the cannabis dosages were controlled using MANCOVA for sexual pain and sexual satisfaction in the COMC, the outcomes were significant, at *p* < 0.05 (see Table 2).

### 3.4. CO Group (n = 17 Participant Responses)

In total, 70.6% of participants felt that their sexual functioning was the same pre- and post-study. However, they felt that using cannabis suppositories had helped them with relaxation (reduced anticipation of pain) during the sexual act (94.1%, n = 16), which increased their sexual connection with their partners (58.8%, n = 10). Participants voiced the need to have more widely available THC suppositories in healthcare (47.1%, n = 8) rather than having to make them up themselves (35.2%, n = 6). When asked about using condoms with cannabis suppositories, limited use was mainly attributed to being in a long-term relationship (52.9%, n = 9) but with some uncertainty about where to obtain oil-resistant condoms (23.5%, n = 4). In terms of using cannabis suppositories, 23.5% (n = 4) cited the use of dilators (sometimes sex toys, including dildos, were used), better sex (23.5%, n = 4) and increased sexual wellbeing, including intimacy and masturbation, were reported (17.5%, n = 3). There appeared to be confusion about using oestrogen gel as a lubricant and concerns about their cancer returning (52.9%, n = 9), with participants often feeling conflicted about whether oestrogen gel was safe to use (47.1%, n = 8).

### 3.5. MC Group (n = 18 Participant Responses)

In total, 72.2% (n = 13) of participants suggested that mindful compassion helped with relaxation and remaining in the moment (83.3%, n = 15), which improved their sexual experiences (77.8%, n = 14). Furthermore, 44.4% (n = 8) reported feeling uncertain about using oestrogen gel but would continue to use their regular lubrication with mindful compassion (27.8%, n = 5). Elsewhere, 22.2 (n = 4) stated that they would consider using mindfulness before using dilators as part of their treatment programme (22.2%, n = 4). In addition, 50.0% (n = 9) felt that it was important for the National Health Service to provide treatment options such as mindful compassion and cannabis suppositories (16.7%, n = 3).

### 3.6. COMC Group (n = 18 of Participant Responses)

In total, 33.3% (n = 6) of participants felt more confident and better after the mindful compassion and cannabis suppository intervention. This was closely followed by feeling more relaxed (27.8%, n = 5). In the group, 50.0% (n = 9) felt that mindful compassion increased the pain-reducing effects of cannabis, which assisted with sexual functioning (33.6%, n = 6). The participants’ lack of condom use was attributed to being in a long-term relationship (50.0%, n = 9), but some felt that there was a lack of information on oil-resistant condoms (41.1%, n = 7). Participants explained that “it’s easy to get CBD suppositories. I mix medical cannabis with CBD suppositories” (72.2%, n = 13), and “I will sometimes mix non-prescribed cannabis oil with CBD suppositories” (27.8%, n = 5). Participants felt that a variety of medical cannabis products should be available in NHS practice (77.8%, n = 14).

### 3.7. CAU Group (n = 16 of Participant Reponses)

There were no differences in sexual functioning for this group. All participants understood their role as the control group and were keen to attend an experimental group post-research study. The team offered mindful compassion group sessions after completing the research to ensure that all groups experienced some perceived improvement. The CO group was offered the same opportunity and appeared content with using cannabis only but was keen to be part of an experimental group in the future.

All groups were asked, “If you had a message to the healthcare team and the team in this study about your treatment regarding sex, what would that be?”.

Based on all the group’s participant responses, n = 54, participants raised the issue of dilators for sexual pain. They suggested that more experimentation with sex toys would be preferable and that perhaps this should have been part of this study’s intervention (38.9%, n = 21). They suggested that gynaecological cancer had diminished their sense of womanhood and sexuality. Dilators, being a medicalised intervention for sexual difficulties, further took away their feeling of sexual attractiveness; hence, they did not engage in standard treatment involving dilators (33.3%, n = 18). They also requested that sexual intimacy be discussed more readily in healthcare as it felt like “a taboo subject” (16.7%, n = 9). Some participants stated that the current study did not use many pictorial examples of female genitalia, which would have been very useful for how “we reconnect with this part of our body, along with the use of sex toys and sexual intimacy with our partners” (11.1%, n = 6).

## 4. Discussion

This study aimed to generate an understanding of how a brief online group-based mindful compassion intervention, combined with cannabis suppositories, might support sexual function and QOL among women after gynaecological cancer. The hypotheses were partially supported. For example, the hypotheses stated that there would be a significant effect of time on sexual self-efficacy, mindful compassion, sexual functioning, well-being and QOL among groups using cannabis suppositories and/or mindful compassion. This hypothesis was supported, but there were variations in how mindful compassion and cannabis suppositories impacted these variables where mixed sexual function outcomes were reported. Overall, the CAU group was non-significant; however, well-being and sexual self-efficacy diminished. Post hoc tests revealed differences across groups with sexual function, sexual-self efficacy, well-being and mindful compassion with the CAU group, compared to the CO, MC and COMC groups.

### MC and COMC Groups

The CO group experienced a reduction in sexual pain, but no other sexual functioning variable was significant. When comparing the outcomes with previous work [24], sexual functioning in a group of 52 MSM levels of sexual pain did not change significantly in the cannabis suppository-only group over 12 weeks. The current sample was larger and, compared to the previous study, more information was available on participants’ cannabis suppository dose and frequency of use, along with anatomical differences. As seen in a previous work [23], levels of well-being and sexual self-efficacy increased from weeks 0 to 12 when using cannabis.

The MC group reported higher levels of sexual arousal, lubrication and orgasms post-intervention. Similarly to a study looking at an online group intervention for women with sexual desire and arousal difficulties, the outcomes showed increased levels of mindfulness post-intervention at 11 weeks [8,35]. Additionally, the effect sizes for lubrication and orgasm were smaller than for sexual arousal. However, compared to Brotto’s [35] outcomes, pain levels did not decrease over time in the current study. The participants in this study stated that having their partners involved in the delivery of the mindfulness intervention might improve overall sexual functioning. Similarly, levels of sexual pain among MSM in the MC group did not diminish over time [24]. Indeed, the outcomes in this study are contrary to outcomes in which mindfulness has consistently been shown to reduce sexual pain [15,36]. However, as seen in a previous study [14], well-being and QOL improved.

The outcomes favoured the COCM group in which sexual function, levels of sexual arousal, lubrication, and orgasm increased, and levels of sexual pain decreased at week 12. Similar outcomes were reported among MSM for sexual pain in the combined cannabis suppository and mindful compassion group. Additionally, mindful compassion, sexual self-efficacy and QOL improved post-intervention [16,24,28].

Mindfulness has been shown to increase levels of sexual self-efficacy, well-being, sexual functioning and QOL [14,16,24,28]. A relationship has been established between sexual self-efficacy and improved sexual functioning and relationship connection [37]. Early menopause was evident in some of the participants in this study. In a study looking at the effects of mindfulness on sexual self-efficacy and sexual satisfaction among n = 110 postmenopausal Iranian women (with n = 55 allocated to the mindfulness experimental group and n = 55 to a control group with no mindfulness intervention over 8 weeks), outcomes favoured the mindfulness group, who showed increased levels of sexual functioning and sexual self-efficacy [38].

Regarding cannabis and sex, THC might act as a vasodilator, allowing more blood flow to the vagina, increasing sexual pleasure [39]. When comparing suppository use with THC, CBD or combined THC/CBD, the combined CBD and THC treatment was favoured among participants in this study. However, this might be owing to the limited availability of THC suppositories in healthcare, such as from the NHS. Therefore, this may have artificially increased the dose to 1000 mg. To elucidate, participants would combine separately obtained THC (e.g., cannabis oil) with ready-made CBD suppositories. Interestingly, 500 mg of THC was also favoured over CBD (500 mg). The outcomes still favoured THC and CBD-combined suppositories to address sexual pain when dosages were controlled. However, further research involving randomisation to sham and controlled dose groups would provide better insight into the dosages required for sexual intimacy and dosage use with dilators. Comparable research is scarce; however, the outcomes looking at THC/CBD among MSM with anodyspareunia were non-significant, possibly due to the small sample size and a lack of information about dosages [24].

The use of suppositories is not a new way of delivering medication. One study [40] evaluated the safety of increasing doses of THC rectal suppositories among male participants and compared the pharmacokinetics of oral administration versus an equivalent amount of rectal suppository, delivered as THC-HS. Outcomes looked promising concerning safety and tolerance. Systemic exposure to cannabis via rectal suppositories (THC) was higher compared to the oral use of THC, where plasma concentrations in rectal suppositories were 2.44-fold higher than with capsule administration. Rectal cannabis suppositories result in reduced feelings of intoxication compared to oral use, as the effects are more localised. In this study, participants suggested that mindful compassion increased the effects of the cannabis suppository. In one study of 47 participants using oral cannabis and engaging in 45 min of yoga, they demonstrated significant improvements in meditation [40]. This finding holds important clinical implications, such as the use of cannabis suppositories to assist with using dilators as part of post-gynaecological cancer treatment. Research is scarce in examining the impact that dilators have on female sexuality, and concerns have been raised about dilators being associated with performance-based sexuality, which could have a detrimental effect [41]. In a qualitative interview-based study of 13 women being treated for vaginismus using an interpretive phenological approach (IPA), 4 superordinate themes were obtained, including a lack of knowledge, invalidation of suffering by professionals, a difficult journey (treatment process) and making the journey easier [42]. Making the journey easier included partner-based and professional support. In the current study, participants referred to the medicalising properties of using dilators and the negative impact that this had on sexuality, resulting in non-engagement. This appeared to be compounded among those who were pre- or post-menopausal. Reference to using sex toys was made, and yet research into using sex toys such as dildos instead of dilators or using vibrators with dilators is scarce. However, NHS Gloucestershire, in the UK, stated that vaginal dilation can be obtained by using sex toys [43]. Those using cannabis suppositories suggested that the use of dilators was easier. While speculatory, the cannabis suppository acted as a lubricant, coupled with pain management.

The limitations of this study are based on ethical and legal cannabis restrictions in the UK. Ideally, medical cannabis should become more readily available on the NHS, including cannabis suppositories. Current NHS prescriptions include epilepsy, chemotherapy, and multiple sclerosis [1]. This limits randomisation to sham and dose-managed groups, along with frequency of use, which would provide a more accurate means of determining the analgesic properties of cannabis. Owing to how the randomisation took place, there was a lack of information on how the suppositories were being used, such as washout. Further, randomisation might have had an anticipatory placebo effect, which impacted participant outcomes [44]. Because controlled randomisation to dose-specific cannabis suppository groups was impossible, owing to the legal restrictions/applications of THC compounds in the UK, the outcomes of this study should be interpreted cautiously. Perhaps a good starting point would be the randomisation of CBD suppositories for sexual pain. Therefore, as a preliminary study, the sample size is adequate, but a larger sample would be required for it to become applicable to the broader population of women with gynaecological cancer. In our study, levels of lubrication had improved. However, this might have been due to an oil-based suppository or to the placebo effect mirrored in the self-report measures [45]. Perhaps the use of a vaginal photoplethysmograph or a Schirmer tear test strip might provide a better understanding. A waiting period for the cannabis suppositories to be absorbed would be needed, as this could give a false positive outcome in the Schirmer test. This would require further research; nonetheless, a novel and potentially more accurate measure of lubrication in participants pre- and post-intervention assessment would be needed [46]. Obtaining support for the current study came with challenges. Therefore, participant recruitment was restricted to social media sites. The research was correct in how randomisation was employed; however, participant bias might have been an outcome [47]. Participants as co-collaborators input into the intervention suggested more pictural representations of the vagina were needed to contextualise the intervention. Plus, there was hesitancy with the use of oestrogen gel as a lubricant or to reduce menopause symptoms, as participants felt conflicted about using oestrogen-based products and their association with cancer (the participant’s perspective). The team predominately consisted of psychologists; a multi-disciplinary approach, including a medical team, would be required for further developing this intervention from a biopsychosocial perspective. Participants felt that their partners would benefit from attending the intervention as using dilators was “non-sexy” without their partners. This may account for the absence of sexual desire outcomes in this study. Therefore, future research might wish to include a relationship/couples-based mindfulness intervention with a psychosexual therapeutic and medical emphasis. The psychosexual education would also include information about oil-resistant condoms as some participants were unaware of their existence, coupled with a lack of condom use. However, the team acknowledged that those participants stated that they were in long-term relationships.

## 5. Conclusions

This exploratory study aimed to establish whether a brief online mindful-compassion-adjunct cannabis suppository group intervention supported women having difficulties with sexual functioning, sexual self-efficacy, well-being and QOL. Both cannabis suppositories and mindful compassion appear to be effective interventions for women after gynaecological cancer treatment with these variables. The cannabis suppositories appeared to address sexual pain, and mindful compassion better mediated the analgesic properties of cannabis to support sexual function, well-being, sexual self-efficacy and QOL. Participants did not engage in using their dilators as part of their treatment, suggesting that the use of sex toys combined with cannabis suppositories and/or mindful compassion might be preferable with their partners. As a preliminary study, the outcomes were informative. This intervention requires development through a biopsychosocial lens and with a larger sample. Ideally, medical cannabis will become more readily available so that researchers can randomly allocate participants to dose-specific THC suppository groups for a better understanding of their potential analgesic properties. Since treatment choice is imperative in healthcare, those who do not wish to use cannabis suppositories may prefer engaging with mindfulness practice, which appeared supportive regarding sexual functioning and QOL. These preliminary outcomes look promising and provide a foundation for future research to develop varied healthcare options to improve mental health service delivery and QOL for this cohort.

## Figures and Tables

**Table 1:** Demographic features of the sample.

		N = 83	
		(n)	%
CO		21	
MC		21	
COMC		21	
CAU		20	
Age			
	18–30	11	13.3
	31–50	51	61.4
	>51	21	25.3
Menopause, including early menopause		43	51.8
Ethnicity			
	White	52	62.7
	African Caribbean	30	36.1
	Pakistani	1	1.2
Sexuality			
	Straight	77	92.8
	Bisexual	6	7.2
Relationship status (years)			
	0–1	7	8.4
	1–2	15	18.1
	3–5	11	13.3
	>5	29	34.9
	Not partnered	21	25.4
Outsider cannabis suppository use, Illicit drug use included			
	None	53	63.9
	Cocaine	7	8.4
	Amphetamine/speed	4	4.8
Outside of post-cancer treatments			
	No medication	44	53
	Herat medication	26	31.3
	Insulin	13	15.7
Alcohol Use			
	None	23	27.7
	<14 units	49	59
	>14 units	11	13.3
Exercise			
	None	42	50.6
	Approximately 3 times per week	15	18.1
	Weekly	18	21.7
	Not stated	8	9.6
Stage of cancer at the time of cancer treatment			
	Stage 1	41	49.4
	Stage 2	39	47
	Stage 3	3	3.6
Type of gynaecological cancer			
	Uterine	43	51.8
	Cervical	30	36.1
	Vaginal	7	8.4
	Vulva	3	3.6
Cancer treatment			
	Surgery	8	9.6
	Radiotherapy	9	10.8
	Chemotherapy	8	9.6
	Combined radiotherapy, chemotherapy and hormones	38	45.8
	Hormones	14	16.9
	Targeted therapy	6	7.2
Use of Cannabis suppositories			
	THC	12	14.5
	CBD	11	13.3
	THC/CBD combined	19	22.9
	Not applicable	41	49.4
Estimated dose of cannabis suppository (mg)			
	100	11	13.3
	500	10	12
	1000	16	19.3
	Unsure	5	6
	Not applicable	41	49.4
Frequency of use of cannabis suppositories			
	Every 2 weeks	22	26.5
	Weekly	13	15.7
	More than weekly	12	14.5
	Not applicable	41	49.4

Key: Cannabis-only group (CO); mindful compassion group (MC). Cannabis suppositories and mindful-compassion group adjuncts (COMC) and care as usual (CAU).

**Table 2:** The means (M) and standard deviations (SD) of the group’s CO, MC, COMC, and CAU before (week 0) and after (weeks 4 and 12) intervention for sexual function, mindful compassion, sexual self-efficacy, well-being and QOL. Significant differences have been included for weeks 0 to 12.

	CO		MC		COMC		CAU	
Week	M	SD	M	SD	M	SD	M	SD
Sexual function								
0	20.29	3.196	22.90	4.134	22.91	4.136	17.60	3.050
4	21.48	3.219	23.24	3.048	23.76	2.965	17.56	2.895
12	21.88 **	3.407	24.440 *	2.064	27.17 **	3.258	16.54	2.670
Sexual desire								
0	2.19	1.030	2.38	0.921	2.33	0.856	1.90	0.788
4	2.38	0.921	2.43	0.870	2.38	0.921	1.89	0.758
12	2.35	0.786	2.56	0.616	2.57	0.659	1.87	0.719
Sexual arousal								
0	4.71	1.554	3.90	1.136	3.91	1.156	4.25	1.164
4	4.67	1.550	4.19	0.928	4.24	0.831	4.33	1.138
12	4.71	1.047	4.61 *	0.788	4.38 *	0.786	4.38	0.957
Lubrication								
0	3.67	1.390	4.00	1.095	4.19	1.03	2.80	1.322
4	3.90	1.179	4.49	1.096	4.19	1.03	2.94	1.259
12	3.88	1.166	4.56 *	1.097	5.00 *	1.029	2.81	1.276
Orgasms								
0	2.05	1.244	2.05	1.244	2.05	1.244	2.80	1.322
4	3.10	1.091	2.19	1.25	2.38	1.284	2.94	1.256
12	3.88	1.166	2.50 *	1.295	2.94 *	1.662	2.81	1.276
Sexual satisfaction								
0	3.10	1.221	4.00	1.871	4.11	1.623	2.80	1.105
4	3.67	1.713	4.05	1.746	4.10	1.609	2.67	1.085
12	3.94	1.952	4.06	0.988	5.00	1.188	2.69	1.138
Sexual pain								
0	6.57	1.832	6.57	1.832	6.83	1.543	6.2	2.042
4	4.00	1.225	6.38	1.396	6.57	1.832	6.22	1.734
12	3.76 **	1.261	6.17	1.2	4.06 *	1.955	6.19	1.759
Mindful compassion								
0	23.24	5.873	23.71	6.597	24.01	5.161	17.50	3.935
4	22.76	5.309	34.10	3.145	36.48	3.669	17.17	3.746
12	22.06	5.771	36.78 **	3.859	37.56 **	3.944	17.00	3.596
Well-being								
0	18.86	4.078	13.33	3.152	13.39	3.162	21.80	3.563
4	22.81	3.487	16.86	3.439	19.67	3.411	19.50	3.015
12	19.41 **	3.709	22.00 **	3.581	23.78 **	3.001	17.50 **	3.141
Sexual self-efficacy								
0	15.19	2.502	15.38	2.156	15.38	2.156	18.00	3.325
4	15.86	2.762	22.33	3.246	23.62	3.413	17.39	2.831
12	17.65 *	3.673	25.39 **	3.032	26.72 *	4.012	15.06 *	2.048
Quality of life								
0	18.81	4.697	17.10	3.048	17.60	4.144	21.60	5.423
4	19.10	4.504	18.86	2.651	19.62	2.889	21.56	4.949
12	20.24	1.505	19.17 *	2.975	19.18 *	2.959	21.31	4.827

* Significant at *p* < 0.05. ** Significant at *p* < 0.001.

## Data Availability

Available upon request from the authors.
